# Effects of neurohormonal antagonists on blood pressure in patients with heart failure with reduced ejection fraction (HFrEF): a systematic review protocol

**DOI:** 10.1186/s13643-020-01452-0

**Published:** 2020-08-24

**Authors:** Rama Krishna Guggilla, Pawel Mateusz Sowa, Jacek Jamiolkowski, Siamala Sinnadurai, Adnan Amin, Karol Adam Kaminski

**Affiliations:** 1 Department of Population Medicine and Civilization Diseases Prevention, Faculty of Medicine with the Division of Dentistry and Division of Medical Education in English, Medical University of Bialystok, ul. Jerzego Waszyngtona 13A, 15-269 Bialystok, Poland; 2 Medical University of Bialystok, ul. Jana Kilinskiego 1, 15-089 Bialystok, Poland

**Keywords:** Heart failure, Treatment, Pharmacotherapy, Reduced ejection fraction, Hypotension, Low blood pressure, Neurohormonal antagonists, Disease-modifying drugs, Guideline-directed medical therapy

## Abstract

**Background:**

Several cardiovascular pathologies cause heart failure. Heart failure with reduced ejection fraction (HFrEF) is deteriorated by neurohormonal activation, so neurohormonal antagonists are recommended in HFrEF patients. They improve morbidity, mortality, and quality of life and reduce hospital admissions. Heart failure treatment guidelines recommend achieving target doses of those drugs. However, many clinicians prescribe suboptimal doses for the fear of inducing hypotension. The aim of this systematic review and meta-analysis is to understand whether it is still beneficial to uptitrate the doses of those drugs even if the patient is at the risk of developing hypotension.

**Methods:**

The primary outcome is symptomatic or asymptomatic hypotension in patients on neurohormonal antagonist drugs for HFrEF. Secondary outcomes are blood pressure reduction, New Yok Heart Association functional class deterioration, non-fatal cardiovascular events, cardiovascular mortality, all-cause mortality, heart failure hospitalizations, and adverse events. Randomized controlled trials involving adults with HFrEF will be included. Comprehensive literature search will be done in MEDLINE, Scopus, Web of Science, WHO Global Index Medicus, and the Cochrane Central Register of Controlled Trials. MEDLINE will be searched first using controlled vocabulary and free text terms and then adapted to other databases. Linear and nonlinear dose-response meta-analyses will be conducted. Publication bias and statistical heterogeneity will be tested by Egger’s regression and Cochran’s Q tests, respectively. Sensitivity, subgroup, and meta-regression analyses will be performed. Grading of Recommendations Assessment, Development and Evaluation approach will be used to judge the quality of evidence.

**Discussion:**

This systematic review and meta-analysis will provide information about the risk of hypotension in patients on neurohormonal antagonist drugs for HFrEF. The results will be published in a peer-reviewed journal. The implications for further research will be discussed.

**Systematic review registration:**

PROSPERO CRD42019140307

## Background

Heart failure (HF) is a progressive, complex clinical syndrome resulting from an abnormality of cardiac structure or function due to myocardial, pericardial, endocardial, valvular, or arrhythmic causes. It leads to reduced cardiac output and/or high filling pressures with exertion or at rest [1]. It is manifested by dyspnea and fatigue leading to exercise intolerance and fluid retention leading to pulmonary congestion and/or peripheral edema [2].

HF is a global health problem affecting an estimated 40 million people worldwide with an incidence of 1–4/1000 person-years [3, 4]. Even though the rates of first hospitalization for HF declined, HF is still one of the leading causes of hospitalization [5]. In 2012, the economic burden of HF was estimated to be around $108 billion per annum, with approximately 60% ($65 billion) being direct costs and the remaining 40% ($43 billion) being indirect costs [6]. HF affects health-related quality of life adversely and dramatically leading to depression, even in their partners [7].

HF is classified based on either the severity of symptoms (New York Heart Association (NYHA) functional classes I–IV) or the level of disease progression (stages A to D of American College of Cardiology Foundation/American Heart Association) [8, 9]. However, for practical purposes, left ventricular ejection fraction (LVEF) is used to distinguish HF into three groups: HF with preserved ejection fraction (HFpEF) with LVEF ≥ 50%, HF with midrange ejection fraction (HFmrEF) with LVEF 40–49%, and HF with reduced ejection fraction (HFrEF) with LVEF < 40% [10].

The goal of HFrEF management, including pharmacological and device therapies, is to reduce morbidity and mortality, improve quality of life, and reduce hospital admissions [11]. Optimal patient outcomes in HFrEF are achieved through guideline-directed medical therapy (GDMT) which addresses pathophysiologic responses in myocardial dysfunction [9]. The mainstay of pharmacological treatment for HFrEF is neurohormonal blockade with the aim to limit disease progression, improve symptoms and quality of life, and reduce mortality [12]. The neurohormonal antagonist drugs used for the treatment of patients with HFrEF include well-established drugs like angiotensin-converting enzyme inhibitors (ACEi) [angiotensin receptor blockers (ARB) if an ACEi is not tolerated], beta blockers (BB), mineralocorticoid receptor antagonists (MRA), and the more recent angiotensin receptor neprilysin inhibitor (ARNI) [13–15]. Other disease-modifying drugs that have shown benefit in HFrEF include hydralazine and isosorbide dinitrate combination, I_f_-channel blocker, and dapagliflozin [16–18]. All the HF clinical practice guidelines recommend treating patients with HFrEF with recommended or maximum tolerated doses of these drugs [9, 19–23]. It has been shown that combination therapy with these drugs improves outcomes than monotherapy [24].

However, it has been found that many patients are not receiving the target doses of these drugs [25–27]. In addition to the beneficial long-term effects on cardiac function, these drugs also have blood pressure-lowering effect [28]. Hypotension is one of the reasons why clinicians do not uptitrate these drugs to their recommended doses [29]. It is a challenging problem in the management of HFrEF. Thus it is very important for clinicians to understand up to what minimum blood pressure levels they can safely uptitrate the guideline-directed medical therapy drugs to maximum doses without increasing morbidity and/or mortality due to hypotension in patients with HFrEF [30]. This systematic review and meta-analysis will attempt to delineate that sweet spot in the management of patients with HFrEF.

## Objective

The objective of this systematic review and meta-analysis is to examine the relationship between neurohormonal antagonist drugs given for the treatment of HFrEF and hypotension. To this end, this systematic review and meta-analysis will specifically address the following three questions:

1. What is the risk of hypotension (blood pressure lower than 90/60 mmHg or mean arterial pressure less than 65 mmHg or presence of specific symptoms such as dizziness, near syncope, and fatigue associated with drop in blood pressure from typical level for a particular patient) in patients on different neurohormonal antagonist drugs given for the treatment of HFrEF?
2. What are the linear and nonlinear dose-response relationships between the neurohormonal antagonist drugs and hypotension?
3. What are the adverse outcomes of treatment with neurohormonal antagonist drugs in patients with HFrEF?

## Methods/design

This protocol has been developed as per the Preferred Reporting Items for Systematic review and Meta-Analysis Protocols (PRISMA-P) 2015 guidelines (Additional file 1) [31].

### Eligibility criteria

#### Study designs

Only randomized controlled trials will be included. Non-randomized controlled trials, controlled before and after studies, prospective and retrospective cohort studies, case-control studies, nested case-control studies, cross-sectional studies, case series, and case reports will be excluded. Non-research articles such as commentaries, letters, and editorials will also be excluded.

#### Participants

Only those studies examining adult (18 years and older) human population diagnosed with HFrEF will be included. The definition of HFrEF is left ventricular ejection fraction < 40%, as per the latest clinical practice guidelines [9, 19, 21–23, 32].

We will include only those studies with participants who were ambulatory with symptomatic heart failure and (1) were randomised to neurohormonal antagonist drugs or placebo (or other inactive substance) or (2) randomised to different doses of same neurohormonal antagonist drugs in the intervention and control arms or (3) different classes of neurohormonal antagonist drugs in the intervention and control arms.

We will exclude those studies in which the comparator was not clearly defined and which were done on people with acute heart failure, hospitalized acute heart failure patients, people with reduced ejection fraction during myocardial infarction, and people with asymptomatic reduced ejection fraction.

#### Interventions

The interventions of interest are neurohormonal antagonist drugs indicated for the pharmacological treatment of HFrEF as recommended by the clinical practice guidelines [9, 19, 21–23, 32]. These include angiotensin-converting enzyme inhibitors (captopril, enalapril, fosinopril, lisinopril, perindopril, quinapril, ramipril, trandolapril), angiotensin receptor blockers (candesartan, losartan, valsartan), mineralocorticoid receptor antagonists (eplerenone, spironolactone), beta blockers (bisoprolol, carvedilol, carvedilol continuous release, metoprolol succinate continuous/extended release, nebivolol), and angiotensin receptor neprilysin inhibitor (sacubitril/valsartan).

#### Comparators

The controls will be the group of people who were given a placebo (or other inactive substance) or another dose of same neurohormonal antagonist drug as in the intervention arm or another class of neurohormonal antagonist drug different from the intervention arm.

#### Outcomes

The primary outcome is symptomatic or asymptomatic hypotension. Asymptomatic hypotension is defined as blood pressure lower than 90/60 mmHg or mean arterial pressure less than 65 mmHg. Symptomatic hypotension is defined as presence of specific symptoms such as dizziness, near syncope, and fatigue associated with drop in blood pressure from typical level for a particular patient.

The secondary outcomes are blood pressure reduction, NYHA functional class deterioration, non-fatal cardiovascular events, cardiovascular mortality, all-cause mortality, heart failure hospitalizations, and adverse events.

For binary outcomes, we will take the number of participants who experienced or not experienced the outcomes in both the intervention and control groups. For continuous outcomes, we will take the number of participants in each of the two arms and the mean and standard deviation of the outcomes. For time-to-event data, we will use the observed and expected values and their variances. We will then calculate the individual study estimates. However, we will extract the estimates of effect directly from individual studies in the case of cluster randomized controlled trials, crossover trials, analyses were adjusted for variables used in stratified randomization or minimization, analysis of covariance was used to adjust for baseline measures of outcome and when time-to-event data is not summarized for each intervention group.

#### Timing

Studies selected for inclusion will not be based on the length of follow-up for outcomes.

#### Setting

There will be no restrictions by type of setting.

#### Language

Only articles reported in English language will be included. If it is feasible to translate articles published in languages other than English using an online or computer-installed machine translation software tool, then other language articles will also be included.

### Information sources

MEDLINE (via both Ovid and PubMed), Scopus, Web of Science, WHO Global Index Medicus, and the Cochrane Central Register of Controlled Trials will be searched.

The electronic bibliographic database search will be supplemented by searching for study records through the ISRCTN registry (http://www.isrctn.com/), WHO International Clinical Trials Registry Platform (http://apps.who.int/trialsearch/Default.aspx), UK Clinical Trials Gateway (https://www.ukctg.nihr.ac.uk/), ClinicalTrials.gov (https://clinicaltrials.gov/ct2/home), EU Clinical Trials Register (https://www.clinicaltrialsregister.eu/ctr-search/search), OpenTrials (https://explorer.opentrials.net/), and metaRegister of Controlled Trials (http://www.isrctn.com/page/mrct).

Grey literature will also be searched through OpenGrey (http://opengrey.org/), MedNar (https://mednar.com/mednar/desktop/en/search.html), Google Advanced Search, and Google Scholar.

### Search strategy

The literature search strategies will be developed using a combination of controlled vocabularies (“exploded” where appropriate) with a wide range of free text terms related to HF and the different classes of neurohormonal antagonist drugs used for the treatment of HFrEF. The specific search strategies will be created by a review team member. The MEDLINE strategy will be developed first by this team member with input from the review team (Additional file 2). After the MEDLINE strategy is finalized, it will be adapted to the syntax and controlled vocabularies (subject headings) of the other databases.

No date limits will be imposed on the search, but the search will be limited to clinical trials, controlled clinical trials, meta-analyses, pragmatic clinical trials, and randomized controlled trials in human subjects only.

To ensure literature saturation, the reference lists of included studies will be scanned for any relevant studies. Forward citation searching will also be used to identify newly published studies.

The literature search will be updated toward the end of the review, in order to capture any additional studies published since the original literature search.

### Selection process

Screening questionnaire for level 1 (title and abstract) and data extraction form for level 2 (full-text articles) will be developed based on the inclusion criteria and will be tested. The two reviewers will independently screen all the titles and abstracts yielded by the literature search against the inclusion criteria. Full-text articles will be obtained for all the titles and abstracts that appear to meet the inclusion criteria and also where there is any uncertainty about eligibility. The two reviewers will then independently screen the full-text articles and decide whether those full-text articles meet the inclusion criteria. Additional information will be sought from study authors, where necessary, to resolve questions about eligibility. Disagreements between the two reviewers will be resolved through discussion between them and, if the disagreements are not resolved through discussion, then through arbitration by a third member of the review team. The reasons for excluding the studies will be recorded. Neither of the two reviewers will be blind to the journal titles or to the study authors or institutions.

### Data management

The abstracts from the literature search, and then the selected full-text articles, will be uploaded to Rayyan web application (https://rayyan.qcri.org/welcome) that facilitates collaboration among the two reviewers during the study selection process [33].

### Data collection

Using the standardized data extraction form, the two reviewers will extract data independently from each study. To ensure consistency across the two reviewers, calibration exercises of data extraction form will be conducted before starting the review. Data extracted will include demographic information, methodology, participant and intervention details, and all patient-important outcomes.

### Data items

The following data will be extracted: study information (title, author, publication year, journal name, author contact details, study funding source, possible conflicts of interest), study characteristics (name of the study, study design, country, setting, inclusion/exclusion criteria, methods of recruitment, duration of follow-up), participant characteristics (sample size, age, gender, race/ethnicity, co-morbidities, other relevant sociodemographics), interventions/comparators and outcomes, statistical methods used for the analysis, risk estimates and 95% confidence intervals, and covariates that were matched or adjusted for in the analysis.

### Risk of bias

To facilitate the assessment of possible risk of bias in each included study, the revised Cochrane Risk-of-Bias tool for randomized trials (RoB 2) will be used to collect information about: randomization process, deviations from intended interventions, missing outcome data, measurement of the outcome, and selection of the reported result [34]. For each domain in the RoB 2 tool, the procedures undertaken for each study will be described. A judgment as to the possible risk of bias in each of the five domains will be made from the extracted information, rated as “low risk of bias,” “some concerns,” and “high risk of bias.”

If sufficient details are not reported in a study, the risk of bias will be judged as “unclear” and then the original study investigators will be contacted for more information.

These judgements will be made independently by the two reviewers based on the criteria for judging the risk of bias. Disagreements between the two reviewers will be resolved first by discussion between them and, if not resolved, then by consulting a third member of the team for arbitration.

Graphic representations of potential bias will be computed within and across studies using the latest major version of Review Manager, which is RevMan 5.3 [35]. Each item in the risk of bias assessment will be considered independently without an attempt to collate and assign an overall score.

### Data synthesis

All statistical analyses will be conducted with “dosresmeta” and “metafor” packages in the “R” software (The R Foundation for Statistical Computing, Vienna, Austria).

The odds ratios and hazard ratios referring to new-onset incident cases of hypotension will be deemed as equivalent to risk ratios (RRs). When neurohormonal antagonist drug dose is illustrated by ranges of intake, the midpoint of the range will be used. When the highest category is open-ended, it will be assumed that the width of the upper category is the same as the adjacent category. When the lowest category is open-ended, the lower boundary will be set to zero.

Two meta-analytical approaches will be undertaken.

Generalized least squares (GLS) will be used to calculate study-specific coefficients across categories of neurohormonal antagonist drug intake accounting for the correlation within each set of RRs. Nonlinear dose-response analysis will be modeled using restricted cubic splines with 3 knots at fixed percentiles (25%, 50%, and 75%) of the distribution. Coefficients estimated within each study will be combined by performing random effects meta-analysis. DerSimonian and Laird’s method will be used for linear dose-response meta-analysis. In nonlinear dose-response meta-analysis, the multivariate extension of the method of moments will be used to estimate the RRs. Whether the two regression coefficients are simultaneously equal to zero will be tested, and a *p* value for nonlinearity will be calculated by testing whether the coefficient of the second spline is equal to zero.

A summary statistic for each study will be calculated, to describe the observed intervention effect (risk ratio if the data are dichotomous and mean difference if the data are continuous). Then a summary (pooled) intervention effect estimate will be calculated as a weighted average of the intervention effects estimated in the individual studies. Both adjusted and unadjusted effect estimates will be included but will be synthesized separately.

A sensitivity analysis will be conducted by excluding one study at a time (leave-one-out), based on study design and quality of evidence, to determine the stability and robustness of the results.

Subgroup analyses by sex, type of neurohormonal antagonist drug, etc. will be performed to test for potential confounders. To further investigate whether other unmeasured potential confounders should be included in the interpretation of the results, the distribution of potential confounders across categories of neurohormonal antagonist drug dose will be investigated. For this purpose, a separate two-stage bivariate meta-analysis for each confounder variable will be performed to determine its association with neurohormonal antagonist drug intake. First, linear regression coefficients (slope and intercept) between neurohormonal antagonist drug intake and the potential confounders will be estimated. Second, GLS will be used to synthesize these intercepts and slope coefficients, accounting for the corresponding variance-covariance matrices.

To determine the significance of the proposed confounders on the association of neurohormonal antagonist drug dose and risk of hypotension, a meta-regression analysis will be conducted. Specifically, the joint slope coefficient of the association will be used as the moderator in the meta-regression analysis.

Publication bias will be assessed using Egger’s regression test, and statistical heterogeneity will be tested by using Cochran’s Q test (statistical significance is defined as a *p* value less than 0.10) and quantified through the multivariate generalization of the *I*^2^ statistic (no, low, medium, and high heterogeneity are defined by *I*^2^ values < 25%, 25–50%, 50–75%, and 75%, respectively).

The quality of evidence for all outcomes will be judged using the Grading of Recommendations Assessment, Development, and Evaluation (GRADE) approach [36]. The quality of evidence will be assessed across the domains of risk of bias, consistency, directness, precision, and publication bias. Additional domains may be considered where appropriate. Quality will be adjudicated as high (further research is very unlikely to change our confidence in the estimate of effect), moderate (further research is likely to have an important impact on our confidence in the estimate of effect and may change the estimate), low (further research is very likely to have an important impact on our confidence in the estimate of effect and is likely to change the estimate), or very low (very uncertain about the estimate of effect).

## Discussion

HFrEF is the final pathway of several different cardiovascular diseases. It poses a major challenge to clinicians in terms of treatment, and a burden to patients and their families in terms of morbidity, mortality, and costs. Even though all the HF clinical guidelines recommend treating patients with HFrEF with target doses of neurohormonal antagonist drugs, it is rarely achieved in routine clinical practice [27, 37]. The fear of inducing hypotension in patients with HFrEF is a major reason for clinicians not to uptitrate those drugs and for patients being reluctant to take increased doses of those drugs.

As per one of the largest surveys across 136 cardiology centers in 12 European countries, the target doses of neurohormonal antagonist drugs are achieved only in about one-third to one-fourth of patients with HF, with the achieved target doses being as low as 16% in case of some drugs [38]. The 1-year follow-up of a prospective, observational registry from 211 cardiology centers in 21 European and/or Mediterranean countries has shown that with the increasing use of neurohormonal antagonists the survival of patients with HF has improved [39]. However, it is interesting to note that the mean baseline systolic blood pressure (SBP) (± standard deviation) of patients with HFrEF in this registry was 121.6 ± 20.0 mmHg with only 34.4% of them with an SBP ≤ 110 mmHg and only 3.9% with peripheral hypoperfusion [40].

This systematic review and meta-analysis will address this major clinical issue in the management of patients with HFrEF by trying to identify the maximum doses of neurohormonal antagonist drugs at which patients develop hypotension. The results of this study will also help us understand the consequences of hypotension in patients with HFrEF. We are not aware of any published or ongoing systematic review and/or meta-analysis addressing the same clinical problem. We expect that the results will help clinicians to uptitrate the neurohormonal antagonist drugs in patients with HFrEF safely unto the maximum doses without inducing hypotension in them. The patients with HFrEF will also be able to make informed decisions about their treatment.

There are several strengths and limitations to our planned methodology. This systematic review and meta-analysis is designed according to the PRISMA guidelines [41, 42]. We want to include only controlled trials, so we may miss some useful information from observation studies. We intentionally developed a very comprehensive search strategy without any limits so as not miss any published literature. However, we will not be able to search all the bibliographic databases because of lack of access to some of them. As we want to include various neurohormonal antagonist drugs used for HFrEF, we expect some potential heterogeneity but we plan to use random effects model approach and do subgroup analyses.

## Supplementary information

**Additional file 1.** PRISMA-P checklist.

**Additional file 2.** MEDLINE (PubMed) search strategy.

**Additional file 3.** Draft data extraction form.

## Data Availability

Not applicable.

## Abbreviations

ACEi: Angiotensin-converting enzyme inhibitor
ARB: Angiotensin receptor blocker
ARNI: Angiotensin receptor neprilysin inhibitor
BB: Beta blocker
GDMT: Guideline-directed medical therapy
GLS: Generalized least squares
GRADE: Grading of Recommendations Assessment, Development, and Evaluation
HF: Heart failure
HFmrEF: Heart failure with midrange ejection fraction
HFpEF: Heart failure with preserved ejection fraction
HFrEF: Heart failure with reduced ejection fraction
ISRCTN: International Standard Randomised Controlled Trials Number
LVEF: Left ventricular ejection fraction
MRA: Mineralocorticoid receptor antagonist
PRISMA-P: Preferred Reporting Items for Systematic review and Meta-Analysis Protocols
PROSPERO: International prospective register of systematic reviews
RoB 2: Revised Cochrane Risk-of-Bias tool for randomized trials
RR: Risk ratio
SBP: Systolic blood pressure
