# Supplementary material for: Effects of neurohormonal antagonists on blood pressure in patients with heart failure with reduced ejection fraction (HFrEF): a systematic review protocol
Source: Syst Rev. 2020 Aug 24;9:194. doi: 10.1186/s13643-020-01452-0 (PMC7445895; doi:10.1186/s13643-020-01452-0)
Supplement: Supplementary file 2 — Additional file 2. MEDLINE (PubMed) search strategy. [file 13643_2020_1452_MOESM2_ESM.docx]

**MEDLINE (via PubMed) Search Strategy**

| **Search** | **Query** |
| --- | --- |
| 1 | "heart failure"[MeSH Terms] |
| 2 | heart failure[Text Word] |
| 3 | 1 OR 2 |
|  | |
| 4 | "angiotensin-converting enzyme inhibitors"[MeSH Terms] |
| 5 | angiotensin converting enzyme inhibitor[Text Word] |
| 6 | ace inhibitor[Text Word] |
| 7 | acei[Text Word] |
| 8 | "captopril"[MeSH Terms] |
| 9 | captopril[Text Word] |
| 10 | "enalapril"[MeSH Terms] |
| 11 | enalapril[Text Word] |
| 12 | "fosinopril"[MeSH Terms] |
| 13 | fosinopril[Text Word] |
| 14 | "lisinopril"[MeSH Terms] |
| 15 | lisinopril[Text Word] |
| 16 | "perindopril"[MeSH Terms] |
| 17 | perindopril[Text Word] |
| 18 | "quinapril"[MeSH Terms] |
| 19 | quinapril[Text Word] |
| 20 | "ramipril"[MeSH Terms] |
| 21 | ramipril[Text Word] |
| 22 | trandolapril[Text Word] |
| 23 | 4 OR 5 OR 6 OR 7 OR 8 OR 9 OR 10 OR 11 OR 12 OR 13 OR 14 OR 15 OR 16 OR 17 OR 18 OR 19 OR 20 OR 21 OR 22 |
| 24 | 3 AND 23 |
|  | |
| 25 | angiotensin ii type i receptor blocker[Text Word] |
| 26 | "angiotensin receptor antagonists"[MeSH Terms] |
| 27 | angiotensin ii receptor blocker[Text Word] |
| 28 | angiotensin receptor blocker[Text Word] |
| 29 | arb[Text Word] |
| 30 | candesartan[Text Word] |
| 31 | "losartan"[MeSH Terms] |
| 32 | losartan[Text Word] |
| 33 | "valsartan"[MeSH Terms] |
| 34 | valsartan[Text Word] |
| 35 | 25 OR 26 OR 27 OR 28 OR 29 OR 30 OR 31 OR 32 OR 33 OR 34 |
| 36 | 3 AND 35 |
|  | |
| 37 | aldosterone receptor antagonist[Text Word] |
| 38 | aldosterone antagonist[Text Word] |
| 39 | "mineralocorticoid receptor antagonists"[MeSH Terms] |
| 40 | mineralocorticoid receptor antagonist[Text Word] |
| 41 | mineralocorticoid antagonist[Text Word] |
| 42 | antimineralocorticoid[Text Word] |
| 43 | "eplerenone"[MeSH Terms] |
| 44 | eplerenone[Text Word] |
| 45 | "spironolactone"[MeSH Terms] |
| 46 | spironolactone[Text Word] |
| 47 | 37 OR 38 OR 39 OR 40 OR 41 OR 42 OR 43 OR 44 OR 45 OR 46 |
| 48 | 3 AND 47 |
|  | |
| 49 | beta adrenoceptor antagonist[Text Word] |
| 50 | beta adrenoceptor blocking agent[Text Word] |
| 51 | beta adrenoceptor blocker[Text Word] |
| 52 | "adrenergic beta-antagonists"[MeSH Terms] |
| 53 | beta adrenergic antagonist[Text Word] |
| 54 | beta adrenergic blocking agent[Text Word] |
| 55 | beta adrenergic blocker[Text Word] |
| 56 | beta blocker[Text Word] |
| 57 | "bisoprolol"[MeSH Terms] |
| 58 | bisoprolol[Text Word] |
| 59 | "carvedilol"[MeSH Terms] |
| 60 | carvedilol[Text Word] |
| 61 | "metoprolol"[MeSH Terms] |
| 62 | metoprolol[Text Word] |
| 63 | "nebivolol"[MeSH Terms] |
| 64 | nebivolol[Text Word] |
| 65 | 49 OR 50 OR 51 OR 52 OR 53 OR 54 OR 55 OR 56 OR 57 OR 58 OR 59 OR 60 OR 61 OR 62 OR 63 OR 64 |
| 66 | 3 AND 65 |
|  | |
| 67 | hydralazine isosorbide dinitrate[Text Word] |
| 68 | 3 AND 67 |
|  | |
| 69 | angiotensin receptor neprilysin inhibitor[Text Word] |
| 70 | arni[Text Word] |
| 71 | sacubitril valsartan[Text Word] |
| 72 | 69 OR 70 OR 71 |
| 73 | 3 AND 71 |
|  | |
| 74 | 24 OR 36 OR 48 OR 66 OR 68 OR 73 |
|  | |
| 75 | 24 OR 36 OR 48 OR 66 OR 68 OR 73 Filters: Clinical Trial; Controlled Clinical Trial; Meta-Analysis; Pragmatic Clinical Trial; Randomized Controlled Trial; Humans |
