# Supplementary material for: Effects of neurohormonal antagonists on blood pressure in patients with heart failure with reduced ejection fraction (HFrEF): a systematic review protocol
Source: Syst Rev. 2020 Aug 24;9:194. doi: 10.1186/s13643-020-01452-0 (PMC7445895; doi:10.1186/s13643-020-01452-0)
Supplement: Supplementary file 3 — Additional file 3. Draft data extraction form. [file 13643_2020_1452_MOESM3_ESM.doc]

**Draft Data Extraction Form**

| Study ID |  |
| --- | --- |
| DOI |  |
| Study author contact details |  |
| Date of data extraction |  |
| Name of person extracting data |  |

# Study eligibility

| Study Characteristics | Eligibility criteria | Eligibility criteria met? | | | | Location in text or source |
| --- | --- | --- | --- | --- | --- | --- |
|  |  | Yes | | No | Unclear |  |
| Type of study | Randomised controlled trial |  | |  |  |  |
| Participants |  |  | |  |  |  |
| Types of intervention |  |  | |  |  |  |
| Types of comparison |  |  | |  |  |  |
| Types of outcome measures |  |  | |  |  |  |
| INCLUDE | | | EXCLUDE | | | |
| Reason for exclusion |  | | | | | |

# Characteristics of included studies

## Methods

|  | Descriptions as stated in report/paper | Location in text or source |
| --- | --- | --- |
| Aim of study |  |  |
| Design |  |  |
| Unit of allocation |  |  |
| Start date |  |  |
| End date |  |  |
| Duration of participation |  |  |

## Participants

|  | Description | | Location in text or source |
| --- | --- | --- | --- |
| Population description |  | |  |
| Setting |  | |  |
| Inclusion criteria |  | |  |
| Exclusion criteria |  | |  |
| Method of recruitment of participants |  | |  |
| Informed consent obtained | Yes No Unclear |  |  |
| Total no. randomised |  | |  |
| Clusters |  | |  |
| Baseline imbalances |  | |  |
| Withdrawals and exclusions |  | |  |
| Age |  | |  |
| Sex |  | |  |
| Race/ethnicity |  | |  |
| Severity of illness |  | |  |
| Co-morbidities |  | |  |
| Other relevant sociodemographics |  | |  |
| Subgroups measured |  | |  |
| Subgroups reported |  | |  |

***Interventions***

|  | Description as stated in report/paper | Location in text or source |
| --- | --- | --- |
| No. randomised to group |  |  |
| Description of intervention |  |  |
| Duration of treatment period |  |  |
| Timing |  |  |
| Co-interventions |  |  |
| Compliance |  |  |

## Outcomes

|  | Description as stated in report/paper | | Location in text or source |
| --- | --- | --- | --- |
| Outcome name |  | |  |
| Time points measured |  | |  |
| Time points reported |  | |  |
| Outcome definition |  | |  |
| Person measuring/ reporting |  | |  |
| Unit of measurement |  | |  |
| Scales: upper and lower limits |  | |  |
| Is outcome/tool validated? | Yes No Unclear |  |  |
| Imputation of missing data |  | |  |
| Assumed risk estimate |  | |  |
| Power |  | |  |

# Data and analysis

Dichotomous outcome

|  | Description as stated in report/paper | | | | | Location in text or source |
| --- | --- | --- | --- | --- | --- | --- |
| Comparison |  | | | | |  |
| Outcome |  | | | | |  |
| Subgroup |  | | | | |  |
| Time point |  | | | | |  |
| Results | Intervention | | | Comparison | |  |
|  | No. with event | Total in group | | No. with event | Total in group |  |
|  |  |  |  |  |  |  |
| Any other results reported |  | | | | |  |
| No. missing participants |  | | |  | |  |
| Reasons missing |  | | |  | |  |
| No. participants moved from other group |  | | |  | |  |
| Reasons moved |  | | |  | |  |
| Unit of analysis |  | | | | |  |
| Statistical methods used and appropriateness of these |  | | | | |  |
| Reanalysis required? | Yes No Unclear | |  | | |  |
| Reanalysis possible? | Yes No Unclear | |  | | |  |
| Reanalysed results |  | | | | |  |

Continuous outcome

|  | | Description as stated in report/paper | | | | | Location in text or source | |
| --- | --- | --- | --- | --- | --- | --- | --- | --- |
| Comparison | |  | | | | |  | |
| Outcome | |  | | | | |  | |
| Subgroup | |  | | | | |  | |
| Time point | |  | | | | |  | |
| Post-intervention or change from baseline? | |  | | | | |  | |
| Results | Intervention | | | Comparison | | |  | |
|  | Mean | SD *(or other variance, specify)* | No. participants | Mean | SD *(or other variance, specify)* | No. participants |  |  |
|  |  |  |  |  |  |  |  |  |
| Any other results reported | |  | | | | |  | |
| No. missing participants | |  | |  | | |  |  |
| Reasons missing | |  | |  | | |  |  |
| No. participants moved from other group | |  | |  | | |  |  |
| Reasons moved | |  | |  | | |  |  |
| Unit of analysis | |  | | | | |  | |
| Statistical methods used and appropriateness of these | |  | | | | |  | |
| Reanalysis required? | | Yes No Unclear | |  | | |  | |
| Reanalysis possible? | | Yes No Unclear | |  | | |  | |
| Reanalysed results | |  | | | | |  | |

Other outcome

|  | Description as stated in report/paper | | | | | Location in text or source |
| --- | --- | --- | --- | --- | --- | --- |
| Comparison |  | | | | |  |
| Outcome |  | | | | |  |
| Subgroup |  | | | | |  |
| Time point |  | | | | |  |
| No. participant | Intervention | | | Control | |  |
|  |  |  |  |  |  |  |
| Results | Intervention result | SE (or other variance) | | Control result | SE (or other variance) |  |
|  |  |  |  |  |  |  |
|  | Overall results | | | SE (or other variance) | |  |
|  |  |  |  |  |  |  |
| Any other results reported |  | | | | |  |
| No. missing participants |  | | |  | |  |
| Reasons missing |  | | |  | |  |
| No. participants moved from other group |  | | |  | |  |
| Reasons moved |  | | |  | |  |
| Unit of analysis |  | | | | |  |
| Statistical methods used and appropriateness of these |  | | | | |  |
| Reanalysis required? | Yes No Unclear | |  | | |  |
| Reanalysis possible? | Yes No Unclear | |  | | |  |
| Reanalysed results |  | | | | |  |

# Other information

|  | Description as stated in report/paper | Location in text or source |
| --- | --- | --- |
| References to other relevant studies |  |  |
| Correspondence required for further study information |  | |
